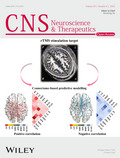# Front cover

**DOI:** 10.1111/cns.14376

**Published:** 2023-07-18

## Abstract

The cover image is based on the Original Article *Multi‐networks connectivity at baseline predicts the clinical efficacy of left angular gyrus‐navigated rTMS in the spectrum of Alzheimer's disease: A sham‐controlled study* by Hai‐Feng Chen et al., https://doi.org/10.1111/cns.14177.